# A rehabilitation program to increase balance and mobility in ataxia of Charlevoix-Saguenay: An exploratory study

**DOI:** 10.1371/journal.pone.0279406

**Published:** 2022-12-28

**Authors:** Isabelle Lessard, Viviane Masterman, Isabelle Côté, Cynthia Gagnon, Elise Duchesne

**Affiliations:** 1 Groupe de Recherche Interdisciplinaire sur les Maladies Neuromusculaires (GRIMN), Centre de Recherche du Centre Intégré Universitaire de Santé et de Services Sociaux du Saguenay–Lac-St-Jean, Québec, Canada; 2 Département des Sciences de la Santé, Université du Québec à Chicoutimi, Québec, Canada; 3 Faculté de Médecine et des Sciences de la Santé, Université de Sherbrooke, Québec, Canada; 4 Centre de Recherche du CHUS de Sherbrooke, Université de Sherbrooke, Québec, Canada; 5 Centre de Recherche Charles-Le Moyne (CRCLM), Université de Sherbrooke, Québec, Canada; Murdoch Children’s Research Institute, AUSTRALIA

## Abstract

Autosomal recessive spastic ataxia of Charlevoix-Saguenay (ARSACS) is characterized by balance impairment and mobility limitations, which both increase the risk of falling. The objective of this study was to explore the effects of a rehabilitation program aimed at increasing trunk and lower limb motor control on balance and walking abilities, and accomplishment of activities of daily living. In this exploratory study, a group-supervised rehabilitation program was performed three times a week for 8 weeks (two sessions at a rehabilitation gym and one pool session). Outcome measures included the Ottawa Sitting Scale, Berg Balance Scale, modified Activities-specific Balance Confidence Scale, 30-Second Chair Stand Test, 10-Meter Walk Test, Barthel Index, and Scale for the Assessment and Rating of Ataxia. Significant improvements in balance, trunk control, maximal and self-selected walking speed difference, ataxia severity and accomplishment of specific activities of daily living were noted for the whole group at the end of the program. At the individual level, all participants improved beyond the standard error of measurement in at least two outcome measures. Also, most participants reported many perceived improvements related to balance, posture and functional mobility. This study provides encouraging results on the effects of a rehabilitation program for ambulatory people with ARSACS. Group intervention could have a positive impact on their daily lives and improve the health care service offered to this population. Future studies with larger sample sizes including control groups and other forms of ataxia are necessary to validate our results to generalize them.

## Introduction

Autosomal recessive spastic ataxia of Charlevoix-Saguenay (ARSACS) is a progressive ataxic disorder [1] caused by mutations in the SACS gene [2] located on chromosome 13q12.12 [3]. Two mutations are mainly present, but over 250 mutations have been identified worldwide [4]. ARSACS is characterized by impairments in three components: pyramidal (spasticity, muscular weakness), cerebellar (ataxia), and distal neuropathy (amyotrophy). Individuals have decreased upper and lower limb coordination, upper limb dexterity, and balance control [5]. They may also experience severe mobility limitations including walking and sit-to-stand transfer limitations, starting in early adulthood and ultimately leading to participation restrictions [5]. Walking abilities are generally lost in their 40s [3, 5]. However, high intersubject variability is observed for most signs and symptoms in terms of severity and progression [5].

To date, there is no cure and only one documented exercise program for ARSACS. The latter was an exploratory study with the objective to improve physical condition using an individualized multimodal program including sports and upper and lower strength training. No effect was observed for physical condition but significant improvement was observed for balance and mobility [6]. In other degenerative ataxias, physical therapy programs have been shown to have positive effects on balance, gait and daily living activities [7, 8]. Another study has also demonstrated that patients with cerebellar ataxia can learn new motor strategies and compensate for their deficits [9]. To the best of our knowledge, no studies have ever focused on a rehabilitation program for the ARSACS population.

The objective of this study was to explore the effects of a rehabilitation program aiming to increase trunk and lower limb motor control and at improving walking abilities, balance and accomplishment of daily living activities (ADLs) in people with ARSACS.

## Methods

### Trial design

An exploratory study.

### Participants

**Fig 1:**
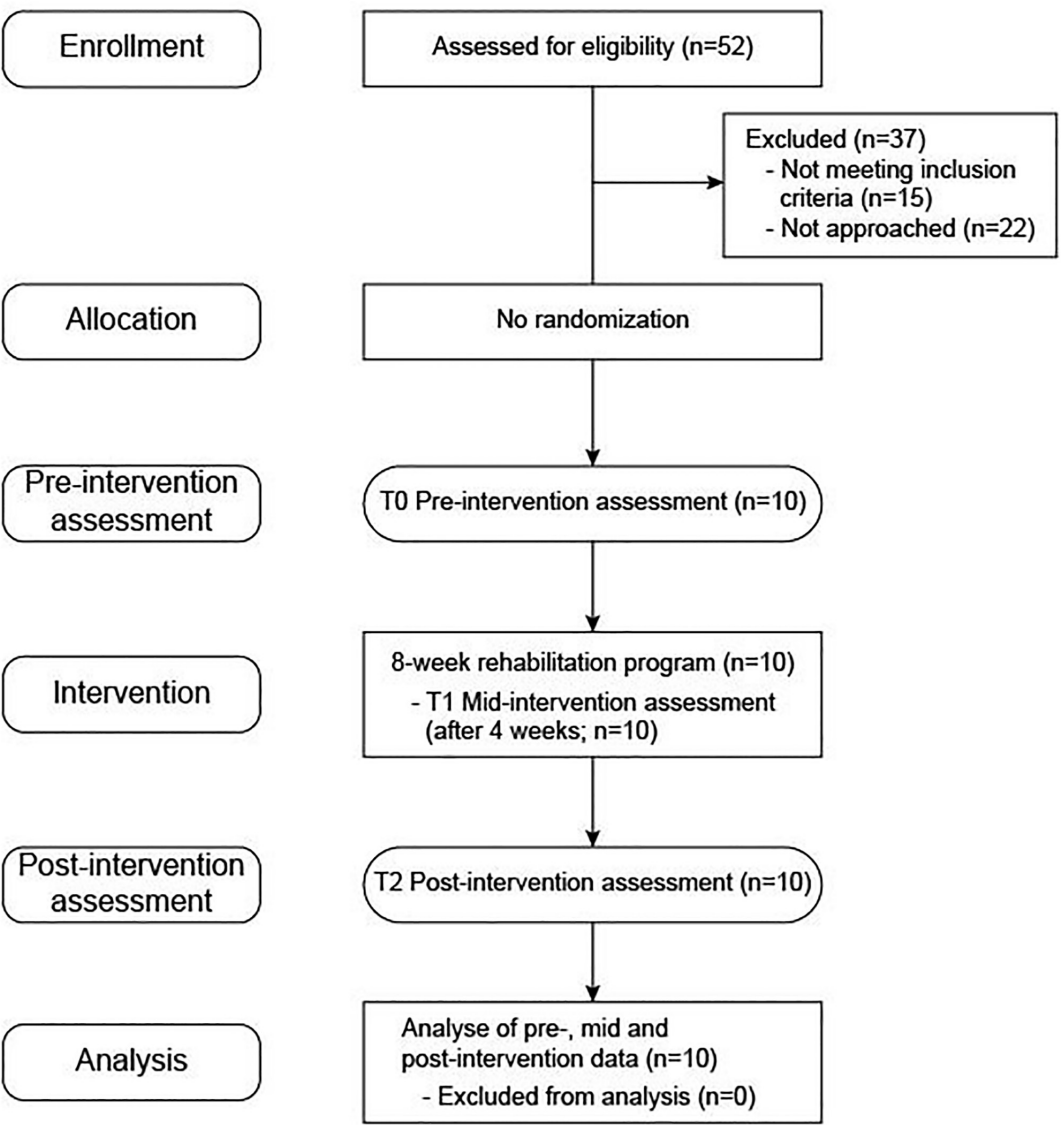
Study flow chart. The diagram illustrates the different stages of the study.

Ten participants were recruited in April 2019 among a subset of 52 people with ARSACS who have participated in a previous longitudinal study led by the *Groupe de recherche interdisciplinaire sur les maladies neuromusculaires*, Québec, Canada (phase 1–2013 [5]; phase 2–2015 [10]; phase 3–2017). They were selected using a convenience sampling strategy. Patients meeting the following criteria were included in the study: (1) ≥18 years old; (2) genetically confirmed ARSACS diagnosis; (3) ability to walk 10 meters (with or without walking aids); (4) ability to provide informed consent. Having other medical diagnosis causing physical limitations was the only exclusion criterion (Fig 1). The Ethics Review Board of the *Centre intégré universitaire de santé et de services sociaux du Saguenay–Lac-St-Jean* (Quebec, Canada) approved the study and written informed consent was obtained. The protocol was registered at clinicaltrials.gov (NCT05479656).

### Intervention

Participants were divided into two groups of five for safety considerations. Two well-trained and experienced physiotherapists (PT) delivered the group rehabilitation program. It involved three training sessions per week (one session [50 minutes] in an adapted pool with 4 levels of water, and two sessions [60 minutes each] at the gym of the rehabilitation center) over 8 weeks, for a total of 24 training sessions. The rehabilitation program focused on the following three domains: 1) postural control, 2) balance, trunk-limbs and multi-joints control and coordination and 3) functional mobility (walking and stairs climbing). Each domain contained a standardized list of exercises with one or more component(s). For each exercise component, different difficulty levels were suggested and standardized. The initial level of difficulty for each exercise was determined by the PT based on individual results of the pre-intervention assessment (T0) related to each domain. To ensure individualized progression throughout the rehabilitation program, the PT ensured high balance challenge for each exercise component based on the participant’s performance, fatigue level and safety (e.g. modifying the level of stabilization or facilitation, reducing the base of support, reducing arm supports, moving to limits of sway, shifting weight, increasing the number of sets and repetitions or diminishing the water level in the pool for the execution of the same exercise). A modality interventions log, including participant adherence, each exercise component with the level of difficulty performed, the number of sets and repetitions (dosage), and the participant’s perceived difficulty, was compiled by the PT at each session for each participant. In the first weeks, the PT taught participants the importance of slowing movement and using good methods of transfers from sitting to standing (anterior displacement of the center of gravity, foot placement, etc.), among other things.

Participants were assessed by the two physiotherapists before the rehabilitation program (T0), after 4 weeks (T1) and after 8 weeks (T2) during a 3-hour session at the *Hôpital de Jonquière* of the *Centre intégré universitaire de santé et de services sociaux du Saguenay–Lac-St-Jean* (Quebec, Canada). Outcome measures were administered following standard operating procedures (SOP) to ensure the reliability of the results between the two raters and the three assessments. The physiotherapists did not assess the same patient at each measurement time point to decrease recall bias. At T0, a sociodemographic questionnaire was administered to document age, sex, height and weight. The type of walking aid used for indoor mobility was also documented at the beginning (T0) and the end (T2) of the project. The genotype of participants was retrieved from their medical records. Using an open-ended question, participants were asked to indicate the main changes they perceived at the end of the program. The adherence rate is based on the number of sessions executed divided by the total number of sessions planned throughout the study.

### Outcomes

#### Trunk control

Sitting balance and trunk control were assessed with the Ottawa Sitting Scale (OSS) [11]. It includes six items graded from 0 to 4, and each item must be done with the feet supported on the ground and unsupported for a total score of 48.

#### Balance

The Berg Balance Scale (BBS) was used to assess balance and risk of falling [12]. It includes 14 items graded from 0 to 4, for a maximum score of 56. Its validity has been demonstrated in ARSACS [13]. The participant’s level of confidence in their balance was assessed using the modified Activities-specific Balance Confidence (m-ABC) Scale. It contains 16 items related to daily activities, each rated from 0% (no confidence) to 100% (completely confident) [14].

#### Mobility

Short-distance walking speed was assessed using the 10-Meter Walk Test (10mWT) at the self-selected and maximal speed. One trial was performed and the speed in meter/second was used for analyses. The 10mWT has shown to be valid in the ARSACS population and has excellent interrater reliability (ICC = 0.992) [13]. The ability to perform sit-to-stand transfers was assessed using the 30-Second Chair Stand Test (30s-CST). The number of completed sit-to-stands performed without support was noted.

#### Activities of daily living (ADL)

The Barthel Index (BI) [15] was administered and includes 10 items and the maximum score is 100.

#### Ataxia severity

The severity of cerebellar ataxia was assessed using the Scale for the Assessment and Rating of Ataxia (SARA), which includes eight items: evaluation of gait, stance, sitting, speech, and four tests assessing dynamic limb function for a total score ranging from 0 (no ataxia) to 40 (most severe ataxia). The inter-rater reliability (ICC = 0.98), test-retest reliability (ICC = 0.90) and internal consistency (Cronbach’s alpha = 0.94) are all excellent in ataxia populations including ARSACS [16, 17].

### Sample size

Given the design (exploratory study), the sample size was based on recommendations for pilot studies [18].

### Statistical analyses

To be included in the statistical analysis, each participant needed to have an adherence rate of at least 80% (at least 17 sessions out of 24) [19, 20]. Descriptive statistics were used: mean, median, SD and ranges for continuous variables, and frequency and percentage for categorical variables. A delta between maximal speed and self-selected speed (Δ 10mWT = maximal speed–self-selected speed) was calculated for T0 and T2. To compare the performance of participants between each assessment (T0, T1 and T2), a Friedman test was used. Post-hoc analyses were done for pairwise comparisons with Bonferroni correction to control for multiple comparisons. Individual change between T2 and T0 was calculated for each outcome measure and compared with the standard error of measurement (SEM), i.e the systematic and random error of a patient’s score that is not attributed to true changes in the construct to be measured [21]. When the SEM was not available for the ARSACS population, the ICC found in the literature for another comparable population and the SD from our sample at T0 were used to calculate the SEM of the measure as follows: SD_T0_*√(1-ICC) [21]. The following SEM values were used in the analysis: BI (2.7) [22], BBS (3.1) [13], 10mWT (0.026) [13], SARA (1.2) [23], 30s-CST (1.27) [24], OSS (1) [11] and m-ABC (4.7) [25]. As used in other exploratory studies, [26–28] p-values <0.1 were a priori selected since they identify clinically relevant for practice improvement, particularly in small studies [29]. Data were analyzed using IBM SPSS Statistics for Mac, Version 20.0 (Armonk, NY: IBM Corp). The open-ended question for participants’ perceived changes at the end of the program was coded using thematic analysis.

## Results

All participants completed the program and had a mean adherence rate of 95% (83–100%). The mean age was 36.6 years old and 60% were women. Table 1 shows all participant characteristics. A total of three participants walked without a walking aid inside their home at T0, and that number increased to five participants after the program (T2).

**Table 1:** Characteristics of the participants.

Age, y		
mean (SD)	36.6 (3.3)	
[min–max]	[30–41]	
Height, cm		
mean (SD)	168.4 (13.0)	
[min–max]	[154.9–190.5]	
Weight, kg		
mean (SD)	76.6 (20.8)	
[min–max]	[49.9–108.9]	
BMI (kg/m^2^)		
mean (SD)	27.0 (6.6)	
[min–max]	[19.5–37.8]	
Sex, n (%)		
Men	4 (40)	
Women	6 (60)	
Genotype, n (%)		
8844delT / 8844delT	8 (80)	
8844delT / 4744G>A	1 (10)	
12992G>A / 12992G>A	1 (10)	
Indoor mobility	Before the program	After the program
Without walking aid	3 (30)	5 (50)
Cane or walker	5 (50)	4 (40)
Wheelchair	2 (20)	1 (10)

BMI: Body mass index.

For the whole group, significant improvements in balance (BBS) (p = 0.067) and sitting balance (OSS) (p = 0.002) were observed between T0 and T2 and in addition for OSS between T1-T2. The change between T0 and T2 is observed in the delta of the walking speed (p = 0.061). The SARA demonstrated a change for two measurement times (T0-T2 and T1-T2) (p = 0.085). A significant change was present for the BI as well for two measurement times (T0-T1 and T0-T2) (p = 0.069). Table 2 shows the mean results of all outcome measures for T0, T1 and T2.

**Table 2:** Performances for T0, T1 and T2.

	Outcomes	Times			p value
		T0	T1	T2	
**Balance**	BBS (/56)	20.5 (10.7)	21.8 (11.6)	22.6 (11.7)	**0.067** ***
	[min-max]	[9.0–38.0]	[11.0–39.0]	[12.0–42.0]	
	OSS (/48)	37.7 (7.5)	40.6 (5.2)	43.3 (4.4)	**0.002** **
	[min-max]	[26.0–48.0]	[29.0–47.0]	[36.0–48.0]	
	m-ABC (/100)	59.3 (10.1)	57.2 (14.1)	63.2 (14.5)	0.798
	[min-max]	[46.7–77.9]	[77.8–57.2]	[46.7–84.4]	
**Mobility**	10mWT s-s				0.905
	T (m/s)	0.51 (0.22)	0.50 (0.26)	0.50 (0.25)	
	[min-max]	[0.27–0.88]	[0.24–1.0]	[0.28–0.91]	
	T (s)	22.76 (8.72)	25.18 (11.53)	23.95 (9.77)	
	10mWT max				0.273
	T (m/s)	0.70 (0.33)	0.72 (0.39)	0.77 (0.42)	
	[min-max]	[0.37–1.45]	[0.40–1.50]	[0.38–1.67]	
	T (s)	16.78 (6.51)	17.08 (7.26)	16.00 (6.40)	
	Δ 10mWT				**0.061** ***
	T (m/s)	0.19 (0.15)	0.22 (0.19)	0.26 (0.21)	
	[min-max]	[0.08–0.57]	[0.08–0.70]	[0.09–0.79]	
	T (s)	5.98 (3.20)	8.10 (5.34)	7.94 (4.60)	
	30s-CST (no. of rep)	1.8 (2.2)	2.3 (2.7)	2.9 (3.1)	0.257
	[min-max]	[0.0–6.0]	[0.0–6.5]	[0.0–7.0]	
**ADLs**	BI (/100)	89.5 (11.2)	93.0 (7.1)	92.0 (10.9)	**0.069** *
	[min-max]	[65.0–100]	[80.0–100]	[65.0–100]	
**Ataxia severity**	SARA (/40)	19.7 (3.0)	18.8 (3.0)	17.8 (3.0)	**0.085** **
	[min-max]	[14.5–23.5]	[13.5–23.0]	[14.0–22.5]	

Results of T0, T1 and T2 are presented as mean (SD).

* Statistical change between T0-T1 and T0-T2 with p <0.10.

** Statistical change between T0-T2 and T1-T2 with p <0.10.

*** Statistical change between T0-T2 with p <0.10.

ADL: Activities of daily living; BI: Barthel index; SARA: Scale for the Assessment and Rating of Ataxia; 10mWT s-s: 10-Meter Walk Test at the self-selected speed; 10mWT max: 10-Meter Walk Test at maximal speed; Δ 10mWT: maximal speed–self-selected speed; 30s-CST: 30-Second Chair Stand Test; m-ABC: modified Activities-specific Balance Confidence; BBS: Berg Balance Scale; OSS: Ottawa Sitting Scale.

All participants showed improvement beyond the SEM in at least two outcome measures (see Table 3). The self-selected walking speed is the variable that improved the least during the program, with only two participants having improved outside the SEM. The mean performance of the whole cohort improved outside the SEM for maximal walking speed, trunk control and disease severity. Sitting balance, maximal walking speed, balance confidence and ataxia severity, are the variables for which the greatest number of participants improved outside the SEM (between 5 and 6). Five participants worsened outside the SEM on a total of one to three outcome measures, including disease severity, balance confidence, walking speed and sit-to-stand transfer.

**Table 3:** Individual change between performances at T2 and T0 for all outcome measures.

Participant ID	ADLs	Ataxia severity	Mobility			Balance			# improved outcomes* (max 8)	# worsened outcomes** (max 8)
	BI	SARA	10mWT s-s	10mWT max	30s-CST	m-ABC	BBS	OSS		
1	+5*	-2*	-0.056**	-0.035**	+1	+8.9*	+2	+3*	4	**2**
2	0	-1	+0.091*	+0.325*	+5*	+15.6*	+4*	0	5	**0**
3	+10*	+1.5**	-0.037**	+0.211*	+3*	+8.9*	-3	+1	4	**2**
4	0	-4.5*	+0.010	+0.104*	+1	+15.6*	+2	+9*	4	**0**
5	+5*	+0.5	0	+0.070*	+1	+13.3*	+1	+6*	4	**0**
6	0	-5.5*	-0.098**	-0.066**	0	-8.9**	+4*	+10*	3	**3**
7	0	-1.5*	+0.027*	+0.013	0	---	+3	+11*	3	**0**
8	0	-3*	-0.017	+0.014	-3**	-2.2	0	+14*	2	**1**
9	+5*	-3*	+0.012	-0.047**	+1	-11.1**	-2	+1	2	**2**
10	0	-0.5	-0.002	+0.218*	+2*	-4.4	+10*	+1	3	**0**
Mean	+2.5	-1.9*	-0.007	+0.065*	+1.1	+4	+2.1	+5.6*	3.4	**1**
*	Improvement outside standard error of measurement (SEM)									
**	Worsening outside SEM									

ADL: Activities of daily living; BI: Barthel index; SARA: Scale for the Assessment and Rating of Ataxia; 10mWT s-s: 10-Meter Walk Test at the self-selected speed; 10mWT max: 10-Meter Walk Test at maximal speed; 30s-CST: 30-Second Chair Stand Test; m-ABC: modified Activities-specific Balance Confidence; BBS: Berg Balance Scale; OSS: Ottawa Sitting Scale.

Table 4 shows the main improvements perceived by the participants at the end of the 8-week rehabilitation program. The answers were grouped into three categories: 1) Balance and posture, 2) Walking capacity/Functional mobility and 3) Others. Among these, improvement of postural control, change or decrease in the use of walking aids and increase in strength are the outcomes most often reported by participants. The most striking finding is the change in walking aids following the program. They also stated that they perceived an improvement in various activities of daily living that required maintaining balance in a standing position, such as taking a shower or requiring good trunk strength, such as wiping after having a bowel movement. Only one participant reported no perceived changes. Although there were decreases in individual performance on some outcomes assessed at the end of the program, no participant reported deterioration in these abilities as a result of participation in the program.

**Table 4:** Perceived improvements reported by participants at the end of the rehabilitation program (n = 9).

Improved functions or activities	n (%)
*Balance and posture*	
Postural control	4 (44.4)
Balance confidence	3 (33.3)
Decrease # of falls	2 (22.2)
Standing posture	1 (11.1)
*Walking capacity/Functional mobility*	
Walking	
Change or decrease in the use of walking aids	4 (44.4)
Walk	2 (22.2)
Increase gait speed	1 (11.1)
Sit-to-stand transfer	3 (33.3)
Activities of daily living	2 (22.2)
Bed mobility	2 (11.1)
Steps	1 (11.1)
*Others*	
Strength (trunk and lower limbs)	3 (33.3)
Daily energy	2 (22.2)
Positive impact on mood	1 (11.1)

## Discussion

This exploratory study suggests the positive effects of an 8-week rehabilitation program aiming to increase trunk and lower limb motor control on walking abilities, balance and accomplishment of daily living activities in ambulatory people with ARSACS.

Significant improvements in balance, trunk control, ataxia severity and accomplishment of specific activities of daily living were noted at end of the program. In addition to the individual improvements outside of SEM observed for many outcome measures, most participants perceived positive effects and reported mainly changes in balance, trunk control, various activities related to mobility and activities of daily living. A recent study showed that an 8-week training program including physical activities, strength power and aerobic training for people with ARSACS (mean age: 28.1 years) has significant benefits on functional mobility activities such as walking (endurance and speed), transfer from sitting to stand, upper and lower limb muscle strength [6]. The results of this previous study are consistent with our findings.

Participants’ results at T0 illustrated the high level of impairments, activity limitations and ataxia severity, which most likely had an impact on the group effects of the rehabilitation program. Only three participants walked without a walking aid inside their home at T0. Two participants used a wheelchair to get around their home, despite being able to walk, due to fear of falling and the need for supervision. The mean age of the participants was 36.6 years, which is close to the age at which walking abilities are lost in this population (38.9 years) [3, 5]. A review suggests that interventions aimed at improving walking abilities in hereditary ataxia populations are more likely to be beneficial earlier in the disease course [30]. Even people with the most advanced disease course could benefit from a rehabilitation program with an improvement in functional abilities. The average improvement of the whole cohort, i.e. 1.9 points on the SARA scale, 2.1 on the BBS and 2.5 on the BI, indicates that the participants regained functional capacity performance equivalent to one year or more of the disease progression [10].

The improvements obtained on the SARA scale for ataxia severity were between T0-T2 and T1-T2, which means that they occurred between five and eight weeks after the start of the rehabilitation program. These results are consistent with the review, which suggests that improvements in ataxia minimally occur after four weeks of intervention [30].

All participants were at high risk of falling at T0 with a BBS score lower than 45 [31] and results lower than 69% on the m-ABC-scale (an indicator of recurrent falls in a similar population) [32]. Despite this high level of impairment, the balance measured using the BBS significantly improved after the program (T0-T2). Fifty percent of the participants showed an improvement outside the SEM for the balance confidence even if the total group did not reach statistical significance. Change or decrease in the use of walking aids reported by four participants are important findings that also reflect balance improvement. However, despite the observed improvements, all participants remained at high risk of falling at the end of the program as demonstrated by the final BBS score.

The most significant improvement following the training program is related to trunk control and seated balance with the OSS. A significant improvement was found between T0-T2 and T1-T2, suggesting that improvements in sitting and standing balance require rehabilitation longer than 4 weeks. The results observed in our study are not in agreement with the review on hereditary ataxia which noted that balance improvement could occur after only three weeks of rehabilitation [30]. At T0, participants had a good static seated balance, but great difficulty maintaining balance when shifting their center of gravity in all directions. A great difficulty in dissociating the trunk movements was also observed in the OSS and this element was greatly improved during the program. Participants perceived positive impacts in carrying out some important activities of their daily life that require the abilities to dissociate the trunk movements, such as wiping after having a bowel movement. The accomplishment of many activities of daily living involves reaching as well as movements of the trunk or upper body to extend the reach.

Decreased trunk control makes it hard to execute sit-to-stand transfers. The performance in mobility activities at each time shows that participants had a lot of difficulties performing the sit-to-stand transfer without using their upper limbs. In fact, their results are similar to those obtained by healthy people over 95 years old [33]. The majority of participants had adaptations in the different rooms of their homes to maintain their autonomy in transfers from sitting to standing. Although the practice of sit-to-stand transfers was part of the program, no significant changes were obtained during the assessment with the 30s-CST test at T1 and T2, and only three participants improved beyond the SEM. This lack of improvement in the number of sit-to-stand transfers achieved in 30 seconds between T0 and T2 could be due to the caution exercised by participants when executing the transfers.

As for the 30s-CST performance, we hypothesize that the lack of significant improvement in the self-selected walking speed was due to the caution exercised by participants, who are very aware of the risk of falling as well as of the importance to have a good walking pattern. Regarding the maximal walking speed, although the change between T0 and T2 is not significant, there is an increase in the maximal average walking speed of the whole group. Additionally, 50% of participants improved their maximal walking speed beyond the SEM. Since the average of the self-selected walking speed did not change, the difference between the maximal and the self-selected walking speed significantly increased between T0 and T2 for the whole group. In other degenerative diseases, such as myotonic dystrophy type 1, this difference between the maximum and self-selected walking speed is a factor that significantly influences the frequency of falls [34]. More precisely, Hammarén et al. [34] showed that an increase in time-difference of 1 s between self-selected and maximal walking speeds, measured over a distance of 10 m, increases the fall risk by 42% in this population. The difference of 1.96 s between the maximal speed and the self-selected speed between T0 (5.98 s) and T2 (7.94 s) supports the importance to develop rehabilitation programs aiming to improve mobility in populations with physical impairments.

Significant improvement in the performance of activities of daily living assessed using the BI was noted between T0-T1 and T0-T2. We hypothesize that the improvements that occurred between T0 and T1 were mainly the result of learning good execution techniques rather than real improvements in functions. As the BI assesses performance in only six basic activities of daily living, it would be interesting for a future study to assess the impact on the performance of a more complete portrait of activities of daily living.

At the end of the training program, deterioration outside the SEM was observed for some participants, mainly in the functional tasks of walking (self-selected [n = 3] and maximal speed [n = 3]) and sit-to-stand transfer (n = 1). People with ARSACS tend to execute their movements very quickly, which impairs the execution quality. In the first weeks of the program, the physiotherapist taught the importance of slowing down the movement speed to increase the execution quality, such as walking and sit-to-stand transfer, to promote good motor learning. It is therefore not surprising that some participants obtained lower scores on tasks that primarily assessed the execution speed, such as the walking speed and the sit-to-stand transfer task. Unfortunately, none of the outcome measures selected were able to validate an improvement in the quality of movement execution in the task. In a future study, it would be interesting to evaluate changes in gait using a layer pressure-sensitive walkway such as the GAITRite.

The most important limitation of this study is the small sample size. To properly document the impact of our rehabilitation program, it would be essential to conduct a study with a larger sample size, including a control group. However, the results of this pilot study help to establish the choice of a primary outcome, as well as the sample size required in future larger studies. In addition, the results of this study and the adherence of the participants support the feasibility and acceptability of rehabilitation programs with this population. As the majority of improvements occurred after the fourth week of intervention, it would be interesting to see the impact of a 12-week program. We also only focused on ambulatory patients and further studies are needed to examine whether people with ARSACS with more severe impairments would also benefit from rehabilitation programs. We also did not evaluate the persistence of gains from this intervention by a follow-up assessment.

The study’s findings provide encouraging results on the effects of a rehabilitation program for ambulatory people with ARSACS. The main findings of this study are the significant improvements in trunk control, balance, and the change in walking aids needed for ambulation. Group rehabilitation programs could have a positive impact on the health care services offered to individuals with ARSACS.

## Supporting information

S1 Checklist CONSORT extension NPT 2017 checklist. (DOCX)

S1 File (DOCX)

## References

[1] Bouchard JP, Barbeau A, Bouchard R, Bouchard RW. Autosomal recessive spastic ataxia of Charlevoix-Saguenay. Can J Neurol Sci. 1978;5(1):61–9. 647499

[2] Engert JC, Berube P, Mercier J, Dore C, Lepage P, Ge B, et al. ARSACS, a spastic ataxia common in northeastern Quebec, is caused by mutations in a new gene encoding an 11.5-kb ORF. Nature genetics. 2000;24(2):120–5. 10655055 10.1038/72769

[3] Bouchard JP, Richter A, Mathieu J, Brunet D, Hudson TJ, Morgan K, et al. Autosomal recessive spastic ataxia of Charlevoix-Saguenay. Neuromuscul Disord. 1998;8(7):474–9. doi: 10.1016/s0960-8966(98)00055-8 9829277

[4] Human Gene Mutation database. 2017 [cited 2016 january 5]. http://www.hgmd.cf.ac.uk/ac/index.php.

[5] Gagnon C, Brais B, Lessard I, Lavoie C, Cote I, Mathieu J. From motor performance to participation: a quantitative descriptive study in adults with autosomal recessive spastic ataxia of Charlevoix-Saguenay. Orphanet J Rare Dis. 2018;13(1):165. doi: 10.1186/s13023-018-0898-z 30231904 PMC6146508

[6] Audet O, Bui HT, Allisse M, Comtois AS, Leone M. Assessment of the impact of an exercise program on the physical and functional capacity in patients with autosomal recessive spastic ataxia of Charlevoix-Saguenay: An exploratory study. Intractable & rare diseases research. 2018;7(3):164–71. doi: 10.5582/irdr.2018.01060 30181935 PMC6119673

[7] Synofzik M, Ilg W. Motor training in degenerative spinocerebellar disease: ataxia-specific improvements by intensive physiotherapy and exergames. BioMed research international. 2014;2014:583507. doi: 10.1155/2014/583507 24877117 PMC4022207

[8] Marquer A, Barbieri G, Perennou D. The assessment and treatment of postural disorders in cerebellar ataxia: a systematic review. Ann Phys Rehabil Med. 2014;57(2):67–78. doi: 10.1016/j.rehab.2014.01.002 24582474

[9] Ilg W, Synofzik M, Brotz D, Burkard S, Giese MA, Schols L. Intensive coordinative training improves motor performance in degenerative cerebellar disease. Neurology. 2009;73(22):1823–30. doi: 10.1212/WNL.0b013e3181c33adf 19864636

[10] Gagnon C, Lessard I, Lavoie C, Cote I, St-Gelais R, Mathieu J, et al. An exploratory natural history of ataxia of Charlevoix-Saguenay: A 2-year follow-up. Neurology. 2018. doi: 10.1212/WNL.0000000000006290 30158165 PMC6177270

[11] Thornton M, Sveistrup H. Intra- and inter-rater reliability and validity of the Ottawa Sitting Scale: a new tool to characterise sitting balance in acute care patients. Disabil Rehabil. 2010;32(19):1568–75. doi: 10.3109/09638280903567893 20662547

[12] Berg K, Wood-Dauphinee S, Williams JI, Gayton D. Measuring balance in elderly: preliminary development of an instrument. Physiother Can. 1989;41:304–11.

[13] Lessard I, Brais B, Cote I, Lavoie C, Synofzik M, Mathieu J, et al. Assessing mobility and balance in Autosomal Recessive Spastic Ataxia of Charlevoix-Saguenay population: Validity and reliability of four outcome measures. J Neurol Sci. 2018;390:4–9. doi: 10.1016/j.jns.2018.03.033 29801904

[14] Powell LE, Myers AM. The Activities-specific Balance Confidence (ABC) Scale. J Gerontol A Biol Sci Med Sci. 1995;50a(1):M28–34. doi: 10.1093/gerona/50a.1.m28 7814786

[15] Mahoney FI, Barthel DW. Functional evaluation: The Barthel Index. Md State Med J. 1965;14:61–5. 14258950

[16] Schmitz-Hubsch T, du Montcel ST, Baliko L, Berciano J, Boesch S, Depondt C, et al. Scale for the assessment and rating of ataxia: development of a new clinical scale. Neurology. 2006;66(11):1717–20. doi: 10.1212/01.wnl.0000219042.60538.92 16769946

[17] Bourcier D, Belanger M, Cote I, Brais B, Synofzik M, Brisson JD, et al. Documenting the psychometric properties of the scale for the assessment and rating of ataxia to advance trial readiness of Autosomal Recessive Spastic Ataxia of Charlevoix-Saguenay. J Neurol Sci. 2020;417:117050. doi: 10.1016/j.jns.2020.117050 32736199

[18] Julious SA. Sample size of 12 per group rule of thumb for a pilot study. Pharmaceutical Statistics. 2005;4(4):287–91.

[19] Williams MT, Lewis LK, McKeough Z, Holland AE, Lee A, McNamara R, et al. Reporting of exercise attendance rates for people with chronic obstructive pulmonary disease: a systematic review. Respirology. 2014;19(1):30–7. doi: 10.1111/resp.12201 24256219

[20] Westcott WL, Winett RA, Annesi JJ, Wojcik JR, Anderson ES, Madden PJ. Prescribing physical activity: applying the ACSM protocols for exercise type, intensity, and duration across 3 training frequencies. The Physician and sportsmedicine. 2009;37(2):51–8. doi: 10.3810/psm.2009.06.1709 20048509

[21] de Vet HCW, Terwee CB, Mokkink LB, Knol DL. Measurement in Medicine: A Practical Guide. Cambridge: Cambridge University Press; 2011.

[22] Hsueh IP, Lee MM, Hsieh CL. Psychometric characteristics of the Barthel activities of daily living index in stroke patients. J Formos Med Assoc. 2001;100(8):526–32. 11678002

[23] Schmitz-Hubsch T, Tezenas du Montcel S, Baliko L, Boesch S, Bonato S, Fancellu R, et al. Reliability and validity of the International Cooperative Ataxia Rating Scale: a study in 156 spinocerebellar ataxia patients. Mov Disord. 2006;21(5):699–704. doi: 10.1002/mds.20781 16450347

[24] Wright AA, Cook CE, Baxter GD, Dockerty JD, Abbott JH. A comparison of 3 methodological approaches to defining major clinically important improvement of 4 performance measures in patients with hip osteoarthritis. The Journal of orthopaedic and sports physical therapy. 2011;41(5):319–27. doi: 10.2519/jospt.2011.3515 21335930

[25] Dal Bello-Haas V, Klassen L, Sheppard MS, Metcalfe A. Psychometric Properties of Activity, Self-Efficacy, and Quality-of-Life Measures in Individuals with Parkinson Disease. Physiother Can. 2011;63(1):47–57. doi: 10.3138/ptc.2009-08 22210979 PMC3024195

[26] Buschert VC, Friese U, Teipel SJ, Schneider P, Merensky W, Rujescu D, et al. Effects of a newly developed cognitive intervention in amnestic mild cognitive impairment and mild Alzheimer’s disease: a pilot study. Journal of Alzheimer’s disease: JAD. 2011;25(4):679–94. doi: 10.3233/JAD-2011-100999 21483095

[27] Hides JA, Franettovich Smith MM, Mendis MD, Smith NA, Cooper AJ, Treleaven J, et al. A prospective investigation of changes in the sensorimotor system following sports concussion. An exploratory study. Musculoskeletal science & practice. 2017;29:7–19. doi: 10.1016/j.msksp.2017.02.003 28259770

[28] Bae JH, Ku B, Jeon YJ, Kim H, Kim J, Lee H, et al. Radial Pulse and Electrocardiography Modulation by Mild Thermal Stresses Applied to Feet: An Exploratory Study with Randomized, Crossover Design. Chinese journal of integrative medicine. 2020;26(4):299–306. doi: 10.1007/s11655-017-2972-0 29150789

[29] Thiese MS, Ronna B, Ott U. P value interpretations and considerations. Journal of thoracic disease. 2016;8(9):E928–E31. doi: 10.21037/jtd.2016.08.16 27747028 PMC5059270

[30] Milne SC, Corben LA, Georgiou-Karistianis N, Delatycki MB, Yiu EM. Rehabilitation for Individuals With Genetic Degenerative Ataxia: A Systematic Review. Neurorehabil Neural Repair. 2017;31(7):609–22. doi: 10.1177/1545968317712469 28595509

[31] Bogle Thorbahn LD, Newton RA. Use of the Berg Balance Test to predict falls in elderly persons. Phys Ther. 1996;76(6):576–85. doi: 10.1093/ptj/76.6.576 8650273

[32] Mak MK, Pang MY. Fear of falling is independently associated with recurrent falls in patients with Parkinson’s disease: a 1-year prospective study. J Neurol. 2009;256(10):1689–95. doi: 10.1007/s00415-009-5184-5 19479166

[33] Rikli RE, Jones CJ. Development and validation of criterion-referenced clinically relevant fitness standards for maintaining physical independence in later years. The Gerontologist. 2013;53(2):255–67. doi: 10.1093/geront/gns071 22613940

[34] Hammaren E, Kjellby-Wendt G, Kowalski J, Lindberg C. Factors of importance for dynamic balance impairment and frequency of falls in individuals with myotonic dystrophy type 1—a cross-sectional study—including reference values of Timed Up & Go, 10m walk and step test. Neuromuscul Disord. 2014;24(3):207–15. 24412157 10.1016/j.nmd.2013.12.003

