# Supplementary material for: A rehabilitation program to increase balance and mobility in ataxia of Charlevoix-Saguenay: An exploratory study
Source: PLoS One. 2022 Dec 28;17(12):e0279406. doi: 10.1371/journal.pone.0279406 (PMC9797069; doi:10.1371/journal.pone.0279406)
Supplement: S1 Checklist — (DOCX) [file pone.0279406.s001.docx]

2017 CONSORT checklist of information to include when reporting a randomized trial assessing nonpharmacologic treatments (NPTs)*. Modifications of the extension appear in italics and blue.

| Section/Topic Item | Checklist item no. | CONSORT item | Extension for NPT trials |
| --- | --- | --- | --- |
| Title and abstract |  |  |  |
|  | 1a | Identification as a randomized trial in the title | N/A, it is a non-randomized trial |
|  | 1b | Structured summary of trial design, methods, results, and conclusions (for specific guidance see CONSORT for abstracts) | Page 2 |
| Introduction |  |  |  |
| Background and objectives | 2a | Scientific background and explanation of rationale | Page 3 |
|  | 2b | Specific objectives or hypotheses | Page 4 |
| Methods |  |  |  |
| Trial design | 3a | Description of trial design (such as parallel, factorial) including allocation ratio | Page 4 |
|  | 3b | Important changes to methods after trial commencement (such as eligibility criteria), with reasons | N/A |
| Participants | 4a | Eligibility criteria for participants | Page 4 |
|  | 4b | Settings and locations where the data were collected | Page 6 |
| Interventions*†* | 5 | The interventions for each group with sufficient details to allow replication, including how and when they were actually administered | Page 5 |
|  | 5a | Description of the different components of the interventions and, when applicable, description of the procedure for tailoring the interventions to individual participants. | Page 5 |
|  | 5b | Details *of whether and* how the interventions were standardized. | Page 5 |
|  | 5c. | Details *of whether and* how adherence of care providers to the protocol was assessed or enhanced | N/A |
|  | 5d | *Details of whether and how adherence of participants to interventions was assessed or enhanced* | Page 8 |
| Outcomes | 6a | Completely defined pre-specified primary and secondary outcome measures, including how and when they were assessed | Pages 6-7 |
|  | 6b | Any changes to trial outcomes after the trial commenced, with reasons | N/A |
| Sample size | 7a | How sample size was determined | Page 4 |
|  | 7b | When applicable, explanation of any interim analyses and stopping guidelines | N/A |
| Randomization: |  |  |  |
| - Sequence generation | 8a | Method used to generate the random allocation sequence | N/A |
|  | 8b | Type of randomization; details of any restriction (such as blocking and block size) | N/A |
| - Allocation concealment mechanism | 9 | Mechanism used to implement the random allocation sequence (such as sequentially numbered containers), describing any steps taken to conceal the sequence until interventions were assigned | N/A |
| - Implementation | 10 | Who generated the random allocation sequence, who enrolled participants, and who assigned participants to interventions | N/A |
| Blinding | 11a | If done, who was blinded after assignment to interventions (for example, participants, care providers, those assessing outcomes) and how | N/A |
|  | 11b | If relevant, description of the similarity of interventions | N/A |
|  | 11c |  | Pages 5-6 |
| Statistical methods | 12a | Statistical methods used to compare groups for primary and secondary outcomes | Page 8 |
|  | 12b | Methods for additional analyses, such as subgroup analyses and adjusted analyses | Page 8 |
| Results |  |  |  |
| Participant flow (a diagram is strongly recommended) | 13a | For each group, the numbers of participants who were randomly assigned, received intended treatment, and were analyzed for the primary outcome | Figure 1 |
|  | 13b | For each group, losses and exclusions after randomization, together with reasons | Figure 1 |
|  | 13c |  | N/A |
|  | new |  | N/A |
| Recruitment | 14a | Dates defining the periods of recruitment and follow-up | Page 4 |
|  | 14b | Why the trial ended or was stopped | N/A |
| Baseline data | 15 | A table showing baseline demographic and clinical characteristics for each group | Page 10 |
| Numbers analyzed | 16 | For each group, number of participants (denominator) included in each analysis and whether the analysis was by original assigned groups | Figure 1 |
| Outcomes and estimation | 17a | For each primary and secondary outcome, results for each group, and the estimated effect size and its precision (such as 95% confidence interval) | Tables 2-3 |
|  | 17b | For binary outcomes, presentation of both absolute and relative effect sizes is recommended | N/A |
| Ancillary analyses | 18 | Results of any other analyses performed, including subgroup analyses and adjusted analyses, distinguishing pre-specified from exploratory | Table 4 |
| Harms | 19 | All important harms or unintended effects in each group (for specific guidance see CONSORT for harms) | N/A |
| **Discussion** |  |  |  |
| Limitations | 20 | Trial limitations, addressing sources of potential bias, imprecision, and, if relevant, multiplicity of analyses | Page 18 |
| Generalizability | 21 | Generalizability (external validity, applicability) of the trial findings | Page 18 |
| Interpretation | 22 | Interpretation consistent with results, balancing benefits and harms, and considering other relevant evidence | Pages 14-18 |
| Other information |  |  |  |
| Registration | 23 | Registration number and name of trial registry | Page 4 |
| Protocol | 24 | Where the full trial protocol can be accessed, if available | Page 4 |
| Funding | 25 | Sources of funding and other support (such as supply of drugs), role of funders | Page 20 |

**Additions or modifications to the 2010 CONSORT checklist. CONSORT = Consolidated Standards of Reporting Trials*

*†The items 5, 5a, 5b, 5c, 5d are consistent with the Template for Intervention Description and Replication (TIDieR) checklist*
