# Supplementary material for: A rehabilitation program to increase balance and mobility in ataxia of Charlevoix-Saguenay: An exploratory study
Source: PLoS One. 2022 Dec 28;17(12):e0279406. doi: 10.1371/journal.pone.0279406 (PMC9797069; doi:10.1371/journal.pone.0279406)
Supplement: S1 File — (DOCX) [file pone.0279406.s002.docx]

**SECTION 1: Team**

Principal investigator:

Cynthia Gagnon

Professeure agrégée - École de réadaptation, Faculté de médecine et des sciences de la santé, Université de Sherbrooke

Directrice du Groupe de recherche interdisciplinaire sur les maladies neuromusculaires

Chercheur régulier - Centre de recherche Charles-Le-Moyne-Saguenay-Lac-St-Jean sur les innovations en santé (CR-CSIS)

Clinique des maladies neuromusculaires, CIUSSS Saguenay-Lac-St-Jean
2230 de l'Hôpital cp 1200
Jonquière, QC
G7X 7X2
418-695-7700 poste 2756
[cynthia.gagnon4@usherbrooke.ca](mailto:cynthia.gagnon4@usherbrooke.ca)

Doctoral student:

Isabelle Lessard

Physical therapist, PhD candidate

**SECTION 2: Title and summary**

A better trunk and lower limb control for a better mobility: Assessment of a re-entrainment program in Autosomal recessive spastic ataxia of Charlevoix-Saguenay

Autosomal recessive spastic ataxia of Charlevoix-Saguenay (ARSACS) is a hereditary neurological disorder presenting with pyramidal (i.e. lower limbs spasticity), cerebellar (i.e. incoordination) and neuropathic (i.e. distal muscle weakness) impairments. Our previous research studies have shown that people with ARSACS have major impairments in regard to upper and lower limbs coordination, upper limbs dexterity, walking speed/endurance and balance control, ultimately leading to participation restrictions and difficulty to perform activities of daily living. No cure exists for ARSACS; at the moment we can only alleviate deficits. However, no study has been published in the scientific literature regarding rehabilitation interventions for this population. Nevertheless, there are some study conducted in degenerative ataxia other than ARSACS which have documented positive effects of physical therapy on balance, gait and performance of daily living activities.

As part of our research program, we have highlighted potential explanatory factors that could be part of a rehabilitation program to improve mobility in this population. We have documented that they have a diminished capacity to move their gravity center (impaired balance control) and to control their lower limbs (synkinesis, co-contraction, incoordination), which as been previously observed in clinical practice. These two phenomena lead to fear of falling of the person when moving forward their gravity center, resulting in severe mobility impairments. As part of a collaboration between the research team and the clinical team, a re-entrainment program designed specifically to increase trunk and lower limb motor control was completed by a 37-year-old woman. This case-study is very promising when comparing results before and after the 6-week program. The participant increased their walking speed, balance, ability to perform sit-to-stand, and respiratory function beyond expectations.

The objective of this pilot project is to document the effects of a re-entrainment program aiming to increase trunk and lower limb motor control on walking capacities, balance and accomplishment of daily activities in people with ARSACS.

This pilot experimental study will use a pre-post design. Participants will be assessed before the intervention (T0), after 4 weeks (T1) and after the 8-week re-entrainment program (T2). Randomized sampling, stratified by age and sex, will be used with the aim to recruit a total of 10 participants. Intervention: The re-entrainment program will be conducted by a physical therapist and either a physiotherapist student/assistant and will consist of 3 sessions per week for 8 weeks. At each week, one session will be performed in the pool, and the two others at the training room. In each session, exercises’ type, order and number of repetitions will be standardized. The 10 participants will be divided into two groups to ensure safety. Participants will need to attend at least 80% of the sessions to be kept in the analysis. Walking speed/endurance, balance, respiratory function, independence level in activity of daily living, and global disease severity will be assessed before, midway and at the end of the program. Participants of our previous natural history study will be prioritized for this project as we will be able to compare the natural progression of the disease with the effects of the program for each participant. Participants’ performance will be individually plotted to illustrate the natural progression of the disease (data obtained in our previous study) against the results obtained just before, in the midway, and after the program for each variable. Exploratory statistical analyses will also be done using Friedman test, a non-parametric test designed for use with repeated measures, to determine if there is a statistical significant improvement following the re-entrainment program.

We aim to have sufficient scientific evidences to convince major granting agencies to fund a larger project, which would include more participants in different neuromuscular clinics to increase the scope of the results and their generalizability. Nevertheless, this grant will bring preliminary evidences that will allow us to begin to inform and convince the medical team about the benefits of physical therapy in this population and maybe to plan to introduce this re-entrainment program at rehabilitation centers.

**SECTION 3: Proposal details**

Autosomal recessive spastic ataxia of Charlevoix-Saguenay (ARSACS) is a progressive devastating hereditary neurological disorder with severe cerebellar and extra-cerebellar brain involvement. The disease is caused by a mutation in the *SACS* gene [1]. Following the recent description of more than 140 known mutations worldwide, it is now described as one of the most common forms of recessive spastic ataxia worldwide [4, 5]. ARSACS is presenting with pyramidal (i.e. lower limbs spasticity), cerebellar (i.e. incoordination) and neuropathic (i.e. distal muscle weakness) impairments. It is a detrimental disorder where individuals present with important activity limitations and participation restrictions including: 1) wheelchair bound around age 33 but with a large unexplained variability (range 19-48) [2]; 2) restrictions related to daily activities after 40 years old where human help is often needed [3]; 3) severe restriction related to work where 70% are unemployed even before age 40 [3]; 4) shortened life expectancy to around 51 years of age [4].

The largest prevalence is found in the Saguenay–Lac-St-Jean region (Quebec, Canada) [5], and our research group (*Groupe de recherche interdisciplinaire sur les maladies neuromusculaires*) conducted the largest interdisciplinary research project to document the natural history of ARSACS over 5 years (2013-18; CIHR/DMC/FAC Emerging Team). Results from the first assessment (2013) have demonstrated major impairments in regard to upper and lower limbs coordination, upper limbs dexterity, walking speed/endurance and balance [6]. Indeed, 21 participants out of 28 have obtained a score below three standard deviations from reference value at the LEMOCOT (lower limb coordination), even in patients before 40 years old (11 out of 16). Only 20% of our participants were within the predicted value for the 10mWT (short distance walking speed) and none for the 6MWT (walking endurance) where all participants obtained between 3 to 66% of the expected value. For balance, 85 % of participants (n=24) were at greater risk of falling (score below 45 at the Berg Balance Scale). We have conducted the second assessment in 2015 and preliminary results with 18 participants are showing a significant decline over the 2-year period: 17% for lower limb coordination, 15% for balance, 21% for walking speed and 13% for walking endurance, and all results were beyond measurement error [7] which support that the decline is a true decline. All these results highlight the need to develop interventions that could help people living with ARSACS by limiting the devastating effects of the disease on their mobility and functional capacity. These results are the backbone of the present grant proposal as they permitted to document the difficulties associated with mobility and highlight some potential explanatory factors. They are the building blocks to develop evidence-based physiotherapy interventions, which is currently lacking in most recessive ataxias even if physiotherapist are highly involved in the clinical care of these populations.

As part of our research program, we have highlighted potential explanatory factors that could be part of a rehabilitation program to improve mobility in this population. We have documented that they have a diminished capacity to move their gravity center (impaired balance control) and to control their lower limbs (synkinesis, co-contraction, incoordination), which as been previously observed in clinical practice [8]. These phenomena lead to fear of falling of the person when moving forward their gravity center, resulting in severe mobility impairments. Clinicians have also observed impaired respiratory functions in this population, which has been poorly documented in the literature. However, association between pulmonary function and trunk muscles has been highlighted in other populations such as stroke [9, 10]. Our doctoral student currently pursuing her Ph.D. on mobility impairments in ARSACS hypothesized that a better motor control of trunk and lower limbs could help people with ARSACS to have a better ability in the sit-to-stand transfer and walking ability, as well as a better respiratory function. Ultimately, this could lead to an improvement of their performance in the activities of daily living where mobility and balance are involved.

To our knowledge, no intervention designed specifically for the ARSACS population has been found in the literature. Nevertheless, a few small-scale studies conducted among individuals with recessive ataxias have documented an improvement in ataxia severity, balance, gait, fall frequency and activities of daily living functioning following physical therapy (e.g. dynamic and static balance control and multi-joint coordination exercises) [11]. Ilg *et al.* have also demonstrated that patients with cerebellar ataxia have the capacity of learning new strategies and to compensate their deficits [12]. A systematic review published in 2017 aimed to evaluate the effects of rehabilitation interventions for individuals with genetic degenerative ataxia [13]. This review suggests that program including more than one focus (such as coordination and balance training) would be more effective than a single focus program. Also, improvement in balance would happen at 3 weeks, and improvement in ataxia within a minimum of 4 weeks. The effectiveness of the program would be greater with an intensity of 60 minutes or greater for 2 days or more per week. It is however important to note that these conclusions are preliminary findings since reviewed studies are heterogeneous in terms of intervention type, intensity, and duration of intervention. Also, none of them have been conducted in ARSACS.

*Preliminary results*

Based on the results found in other degenerative ataxic populations and our research and clinical knowledge of ARSACS, a re-entrainment program designed specifically to increase trunk and lower limb motor control was developed by our research and clinical team. We try this preliminary program (50 minutes, 3 days per week, 6 weeks) with one ARSACS patient followed at the Saguenay Neuromuscular Clinic, who participated in the last two data collections of our previous research project aiming at identifying explanatory factors of mobility problems. The patient is a 37-year-old woman who uses a walker inside and outside her home to walk.

Results are presented in Table 1 and are very encouraging. An improvement of all parameters was found. For respiratory functions, it is important to note that interventions are recommended when the peak expiratory flow is below 270 L/min, and that the minimum ratio to have an effective cough is 1. In this single case study, the participant has increased these two parameters above the critical threshold following the 6-week program. Results are thus highly promising, and this is why we want to extend the number of participants with this grant application. The re-entrainment program proposed here could be easily performed in clinical setting, with a high impact on the quality of life of patients as mobility is among the main issues for this population.

Table 1: Results of the single case study before and after the re-entrainment program

|  | **Before** | **After** | **Improvement (%)** |
| --- | --- | --- | --- |
| **Comfortable walking speed** | 0.52 m/s | 0.61 m/s | 17.3 |
| **Maximum walking speed** | 0.60 m/s | 0.73 m/s | 21.7 |
| **30-second Sit-to-stand** | 2 reps | 5 reps | 150 |
| **Berg Balance Scale** | 22 | 28 | 27.3 |
| **Peak expiratory flow** | 213.3 L/min | 302.5 L/min | 41.8 |
| **Cough ratio** | 0.78 | 0.94 | 20.5 |

Despite the smaller scope of the present grant proposal (10 participants) due to financial costs, this project will be highly innovative and will provide the necessary preliminary data to apply to major funding initiatives such as CIHR in order to provide to healthcare professionals worldwide research results to better informed evidence-based knowledge.

OBJECTIVES
The objective of this pilot project is to document the effects of a re-entrainment program aiming to increase trunk and lower limb motor control on walking capacities, balance and accomplishment of daily activities in people with ARSACS.

The main hypothesis is that participants will improve their walking speed following the re-entrainment program beyond measurement error.

The secondary hypothesis is that participants will improve their performance on the other variables following the re-entrainment program.

METHODOLOGY

**Design.** This pilot experimental study will use a pre-post design. Participants will be assessed before the intervention (T0), after 4 weeks (T1) and after the 8-week re-entrainment program (T2).

**Population.** Participants will be recruited among those who participated to our previous natural history study (n = 52 either assessed in 2015 and/or 2018). In this way, we will be able to compare the progression of the disease over the last years with the intervention’s effects for each participant. If necessary, 10 patients included in the clinical registry of the Saguenay neuromuscular clinic who can walk could be recruited. Inclusion criteria are: 1) to be aged between 18 and 40 year-old, 2) be able to walk inside (with or without walking aid), and 3) to have a genetic diagnosis of ARSACS. Exclusion criteria will be the presence of other medical conditions that can lead to significant functional deficits or multimorbidity preventing the participants to complete the program and/or all tests. **Sampling.** Randomized sampling, stratified by age and sex, will be used with the aim to recruit a total of 10 participants among our previous participants. **Re-entrainment program.** The re-entrainment program will be conducted by a physical therapist and either a physiotherapist student/assistant and will consist of 3 sessions per week for 8 weeks. See Q3 and Q4 documents for the complete program. At each week, one session will be performed in the pool, and the two others at the training room. In each session, exercises’ type, order and number of repetitions will be standardized. The 10 participants will be divided into two groups to ensure safety. Participants will need to attend at least 80% of the sessions to be kept in the analysis. If participants quite the program, we will document the reasons and potential solutions to improve adherence to the program.

|  | **Monday** | **Tuesday** | **Wednesday** | **Thursday** | **Friday** |
| --- | --- | --- | --- | --- | --- |
| **Week 1** | *Pre-program assessment according to participant availability* | | | | |
| **Week 2** | Training room |  | Pool |  | Training room |
| **Week 3** | Training room |  | Pool |  | Training room |
| **Week 4** | Training room |  | Pool |  | Training room |
| **Week 5** | Training room | *Midway assessment* | | | |
|  |  | Participants 1-2 | Participants 3-4-5 | Participants 6-7-8 | Participants 9-10 |
| **Week 6** | Training room |  | Pool |  | Training room |
| **Week 7** | Training room |  | Pool |  | Training room |
| **Week 8** | Training room |  | Pool |  | Training room |
| **Week 9** | Training room |  | Pool |  | Training room |
| **Week 10** | *Post-program assessment according to participant availability* | | | | |

*Pool:* This session will last for 50 minutes. Exercises will aim to improve seated balance, standing balance and posture, strength of trunk and abdominal muscles, motor control of lower limb and trunk, and endurance.

Examples of exercises included in the standardized program: to swim in a seated position (on a float) using upper limbs, half bell (move from lying on the back to standing position, with an intermediate grouped position), walking, standing posture correction, etc.

*Training room:* This session will last 60 minutes. Exercises will aim to improve strength of trunk and motor control of lower limb, to stimulate the movement of the gravity center and reactions of rebalancing.

Examples of exercises included in the standardized program: reaching, sit to stand transfers (with exercise ball), go up and down the stairs, correction of walking with walking aid (short distance), posture correction on parallel bars, etc.

Over the weeks, difficulty of exercises will be increased according to the capacity and improvement of each participant by modifying the stabilization level (e.g. sit in the bed first, then use of an exercise ball), and by increasing the number of repetitions or the water level in the pool.

**Variables and measurement tools.** The selection is based on our previous longitudinal study and their demonstrated metrological properties in ARSACS. All measures will be administered using standard operational procedures. A general questionnaire will be answered at T0 to collect data about age, sex, mobility level, and walking aids used. All other tests and questionnaires will be carried out at T0, T1 and T2. The 10-Meter Walk Test (10mWT) (comfortable and maximum pace) will be used to assess short distance walking speed by measuring the time required to cover a 10-meter distance. The 6-Meter Walk Test (6MWT) will also be used to determine the walking capacity on long distance. The American Thoracic standard guidelines will be followed [14]. Both 10mWT and 6MWT have shown to be valid in ARSACS and have an excellent interrater reliability (ICC = 0.97-0.99) [2]. The 30-Second Chair Stand Test will be used to assess lower lib power. It is the number of stands a person can complete from a chair in 30 seconds. The Lower Extremity Motor Coordination Test (LEMOCOT) will be used to assess coordination [15, 16]. The Berg Balance Scale [17] will be used to assess balance and fall risk. It includes 14 items graded from 0 to 4, for a maximum score of 56 (higher values indicate better performance). Its validity has been demonstrated in ARSACS [2]. Also, the fear of falling will be determined using the modified Activities-specific Balance Confidence (ABC) Scale. It contains 16 items related to daily activities, each rated from 0% (no confidence) to 100% (completely confident) [18]. The Scale for the Assessment and Rating of Ataxia (SARA) will be used as global disease severity measure. It includes eight items: evaluation of gait, stance, sitting and speech, and four tests assessing dynamic limb function. The inter-rater reliability (ICC=0.98), the test-retest reliability (ICC=0.90) and the internal consistency (Cronbach’s alpha = 0.94) were all high [19]. The Ottawa sitting scale will be used to characterise sitting balance [20]. It includes 12 items graded from 0 to 4, for a maximum score of 48 (higher values indicate better performance). The peak expiratory flow (PEF) and the cough peak flow (CPF) will be measured using the Airlife^TM^ Asthma Check peak flow meter. The ratio CPF / PEF will then be used to determine the glottis efficacy. A ratio value under 1 indicates an ineffective glottis. The Barthel Index [21] will be used to measure the level of independence level in activity of daily living.

**Data analysis.** Descriptive analysis will be performed for each variable at each measurement time. Participants’ performance will be individually plotted to illustrate the natural progression of the disease (data obtained in our previous study) against the results obtained just before, in the midway, and after the program for each variable. Exploratory statistical analyses will also be done using Friedman test, a non-parametric test designed for use with repeated measures, to determine if there is a statistical significant improvement following the re-entrainment program.

DELIVERABLES

With this project, we aim to have sufficient scientific evidences to convince major granting agencies to fund a larger project, which would include more participants in different neuromuscular clinics to increase the scope of the results and their generalizability. Nevertheless, this grant will bring preliminary evidences that will allow us to begin to inform and convince the medical team about the benefits of physical therapy in this population and maybe to plan to introduce this re-entrainment program at rehabilitation centers. We strongly believe that it can help individuals living with ARSACS to have a better quality of life and more easily perform activities of daily living.

**Knowledge translation plan.**

*Research and Clinical communities:* The results will be submitted to an academic journal. In addition, results will be presented at the next International ataxia research conference in 2019 and at the MDC congress in 2021. The results will permit to develop a wiki page to post on the CAN-NMD wiki website for clinicians.

*Patients and families:* We will contact MDC to organize a webinar for both patients/families with ARSACS. We will also include our team of expert patients to define appropriate communication strategies for patients and families.

**BUDGET**

**Total budget requested: 40 639,65$**

One physical therapist will be hired to conduct the intervention with participants. As a university clinic, the Saguenay Neuromuscular Clinic welcomes physical therapist trainees three times a year. We will carry out this project at the same time than a traineeship to decrease staff remuneration costs while offering security and expertise to the participants. In this way, we will have two additional physical therapists to conduct the intervention. A research coordinator will also be hired for the ethical approval, and the schedule/budget management. A research professional will be hired for the recruitment of participants and follow-up with them (appointment reminder) to increase the compliance with the program. At the end of the project, the research professional will be responsible of the data entry, statistical analyses, and writing of the scientific paper. Finally, a financial compensation for transportation fees will be given to participants at each visit.

*Physical therapist*

(23 sessions X 4hrs/session) + (10 participants X 3 assessments X 2hrs/assessment) =

152hrs X 58.95$* = 10 728,90$

*Research coordinator*

50hrs (ethical approval) + (7hrs / week X 10 weeks) =

120hrs X 52.40$* = 6288$

*Research professional #1*

1hr / participant (recruitment) + 0.25hr / participant X 26 sessions (appointment reminder) =

97,5hrs X 39.30$* = 3 837,75$

*Research professional #2*

1hr X 15 participants (data entry) + 35hrs (data analyses) + 4 weeks X 35hrs (publication writing) =

190hrs X 45$* = 8550$

*Participant fees*

15 participants X 26 sessions X 20$/session = 7785$

*Material*

Flow meter 🡪 15 X 30$ = 450$

Knowledge translation material = 3000$

Production of a communication strategy for the participants and the larger patients’ community based on our work with our expert patients. As we cannot anticipate the best format to deliver the information at this moment based on previous projects we ask for 3000$. An expert patient will come with us at one of the congress to present the results. In addition, we have a lunch-type activity with participants to share the results of the study. In addition, we will like to produce a short video explaining the results of the study for our interactive waiting room at the Neuromuscular clinic and that could be posted on MDC website.

* *Hourly rate including benefits*

**References**

[1] Engert JC, Berube P, Mercier J, Doré C, Lepage P, Ge B, et al. ARSACS, a spastic ataxia common in northeastern Quebec, is caused by mutations in a new gene encoding an 11.5-kb ORF. Nat Genet 2000;24:120-5.

[2] Lessard I, Brais B, Côté I, Lavoie C, Synofzik M, Mathieu J, et al. Assessing mobility in Autosomal Recessive Spastic Ataxia of Charlevoix-Saguenay population: Validity and reliability of four outcome measures. J Neurol Sci 2018;390:4-9.

[3] Gagnon C, Brais B, Lessard I, Lavoie C, Côté I, Mathieu J. Social participation and activities of daily living in Autosomal Recessive Spastic Ataxia of Charlevoix-Saguenay. *In preparation*.

[4] Dupre N, Bouchard JP, Brais B, Rouleau GA. Hereditary ataxia, spastic paraparesis and neuropathy in the French-Canadian population. Can J Neurol Sci 2006;33:149-57.

[5] Bouchard JP, Barbeau A, Bouchard R, Bouchard RW. Autosomal recessive spastic ataxia of Charlevoix-Saguenay. Can J Neurol Sci 1978;5:61-9.

[6] Gagnon C, Brais B, Lessard I, Lavoie C, Côté I, Mathieu J. From motor performance to participation: A quantitative descriptive study in adults with Autosomal recessive spastic ataxia of Charlevoix-Saguenay. Orphanet J Rare Dis 2018;13:165.

[7] Gagnon C, Brais B, Lessard I, Lavoie C, Côté I, St-Gelais R, et al. An exploratory natural history of Ataxia of Charlevoix-Saguenay: A two-year follow-up. Neurology 2018;Epub ahead of print.

[8] Cashman-Kadri J-E, Hébert L, Lessard I. Caractérisation des profils de co-activations musculaires au membre inférieur des personnes atteintes d'ataxie récessive spastique de Charlevoix-Saguenay lors de mouvements simples; étude de faisabilité. in Colloque étudiants du Centre interdisciplinaire de recherche en réadaptation et intégration sociale. 2017: Québec, Canada.

[9] Lee K, Cho JE, Hwang DY, Lee W. Decreased Respiratory Muscle Function Is Associated with Impaired Trunk Balance among Chronic Stroke Patients: A Cross-sectional Study. Tohoku J Exp Med 2018;245:79-88.

[10] Lee DK, Kim SH. The effect of respiratory exercise on trunk control, pulmonary function, and trunk muscle activity in chronic stroke patients. Journal of physical therapy science 2018;30:700-3.

[11] Marquer A, Barbieri G, Perennou D. The assessment and treatment of postural disorders in cerebellar ataxia: a systematic review. Ann Phys Rehabil Med 2014;57:67-78.

[12] Ilg W, Synofzik M, Brotz D, Burkard S, Giese MA, Schols L. Intensive coordinative training improves motor performance in degenerative cerebellar disease. Neurology 2009;73:1823-30.

[13] Milne SC, Corben LA, Georgiou-Karistianis N, Delatycki MB, Yiu EM. Rehabilitation for Individuals With Genetic Degenerative Ataxia: A Systematic Review. Neurorehabil Neural Repair 2017;31:609-22.

[14] American Thoracic Society. ATS statement: guidelines for the six-minute walk test. Am J Respir Crit Care Med 2002;166:111-7.

[15] Desrosiers J, Rochette A, Corriveau H. Validation of a new lower-extremity motor coordination test. Arch Phys Med Rehabil 2005;86:993-8.

[16] Lessard I, Lavoie C, Côté I, Mathieu J, Brais B, Gagnon C. Validity and reliability of the LEMOCOT in the adult ARSACS population: A measure of lower limb coordination. Journal of the Neurological Sciences 2017;377:193-6.

[17] Berg K, Wood-Dauphinee S, Williams JI, Gayton D. Measuring balance in elderly : preliminary development of an instrument. Physiother Can 1989;41:304-11.

[18] Powell LE, Myers AM. The Activities-specific Balance Confidence (ABC) Scale. J Gerontol A Biol Sci Med Sci 1995;50a:M28-34.

[19] Schmitz-Hubsch T, du Montcel ST, Baliko L, Berciano J, Boesch S, Depondt C, et al. Scale for the assessment and rating of ataxia: development of a new clinical scale. Neurology 2006;66:1717-20.

[20] Thornton M, Sveistrup H. Intra- and inter-rater reliability and validity of the Ottawa Sitting Scale: a new tool to characterise sitting balance in acute care patients. Disabil Rehabil 2010;32:1568-75.

[21] Mahoney FI, Barthel DW. Functional evaluation: The Barthel Index. Md State Med J 1965;14:61-5.
